# HIV Prevention Services and Testing Utilization Behaviors among Men Who Have Sex with Men at Elevated Risk for HIV in Chongqing, China

**DOI:** 10.1155/2014/174870

**Published:** 2014-03-26

**Authors:** Dayong Huang, Yifei Hu, Guohui Wu, Yujiang Jia, Rongrong Lu, Yan Xiao, H. F. Raymond, Willi McFarland, Yuhua Ruan, Wei Ma, Jiangping Sun

**Affiliations:** ^1^Beijing Friendship Hospital, Capital Medical University, Beijing 100050, China; ^2^National Center for AIDS/STD Control and Prevention, Chinese Center for Disease Control and Prevention, 155 Changbai Road, Changping District, Beijing 102206, China; ^3^Chongqing Center for Disease Control and Prevention, Chongqing 400042, China; ^4^Department of Preventive Medicine, Vanderbilt University School of Medicine, Nashville, TN 37232, USA; ^5^San Francisco Department of Public Health, San Francisco, CA 94102, USA; ^6^Department of Epidemiology and Biostatistics, University of California, San Francisco, CA 94105, USA; ^7^State Key Laboratory for Infectious Disease Prevention and Control, National Center for AIDS/STD Control and Prevention, Chinese Center for Disease Control and Prevention, Collaborative Innovation Center for Diagnosis and Treatment of Infectious Diseases, Beijing 102206, China; ^8^Shandong University School of Public Health, Jinan 250012, China

## Abstract

*Objective*. To investigate barriers and correlates of the use of HIV prevention services and HIV testing behaviors among men who have sex with men in Chongqing. *Methods*. Three consecutive cross-sectional surveys provided demographic, sexual behavior, HIV/syphilis infection, HIV prevention service, and testing behavior data. *Results*. Of 1239 participants, 15.4% were infected with HIV, incidence was 12.3 per 100 persons/year (95% CI: 9.2–15.3), 38% of the participants reported ever having unprotected insertive anal sex, 40% ever received free condom/lubricants in the past year, and 27.7% ever obtained free sexually transmitted infection examination/treatment in the past year. Multivariable logistic regression revealed that lower levels of HIV/AIDS related stigmatizing/discriminatory attitudes, full-time jobs, and sex debut with men at a younger age were independently associated with use of free condom/lubricants. Large social networks, higher incomes, and sexual debut with men at a younger age were associated with use of any HIV prevention and HIV testing services. Lower levels of stigmatizing/discriminatory attitudes were also associated with HIV testing. Fearing needles and being unaware of the venues for testing were top barriers for testing service utilization. *Conclusion*. It is imperative to address HIV/AIDS related stigmatizing/discriminatory attitudes and other barriers while delivering intervention and testing services.

## 1. Introduction

In recent years, the HIV epidemic has been rapidly expanding among men who have sex with men (MSM) in China. Male-to-male sexual contact has emerged as one of the major transmission routes for new HIV infections [1–3]. Historically, HIV-1 infection has been largely concentrated among injection drug users and former paid blood and plasma donors in geographically disparate rural areas in China [4, 5]. However, the rapid increase of HIV-1 prevalence among MSM [6, 7] shows that China may be following the trajectory of other Asian countries where HIV-1 infection is a growing problem among MSM [3, 8–10].

Chongqing is one of the metropolitan cities in China with a rapid expansion of the HIV epidemic among MSM [11]. HIV prevalence among MSM in Chongqing [12] was twice the rates of 5-6% among MSM from the National Sentinel Surveillance in 2011 [13]. A meta-analysis of HIV studies among MSM in low and middle-income countries estimated that globally MSM have 19.3 times higher odds of being infected with HIV compared to the general population [14]. Among an estimated 780,000 persons living with HIV/AIDS in China, nearly 55% did not know their infection status in 2011 [3]. A recent study in Shanghai reported 28.5% of MSM engaged in sex work (money boys) and 50.5% of non-money boy MSM had never tested for HIV [15]. It is imperative to effectively reach MSM and improve the effectiveness and quality of ongoing intervention prevention services and promotion of HIV testing [16].

HIV prevention services, including the distribution of condoms, HIV testing, and testing and treatment for sexually transmitted infections (STIs), have been documented as effective measures in reducing HIV transmission [17–21]. HIV testing is the entry point to access other interventions and treatment services, while HIV infected individuals who know their infection status have been shown to reduce their risky sexual behavior and lower the risk of onward transmission [17, 19]; moreover timely treatment of HIV infected individuals may reduce community level viral load and prevent HIV transmission at the population level [22, 23].

Though HIV prevention services and HIV testing have been scaled up in China over the past few years, gaps in the use of these services still remain [24–26]. Limited data is available in characterizing barriers and facilitators for the use of these prevention services in different settings or regions [16].

From 2009 to 2013, three consecutive cross-sectional surveys were conducted among MSM in Chongqing using respondent driven sampling (RDS). The objective of this study was to investigate the barriers and correlates of HIV prevention services and testing behaviors among MSM in Chongqing.

## 2. Methods

### 2.1. Recruitment of the Participants

Three consecutive cross-sectional surveys were conducted among MSM from September of 2009 to January 2010, September 2010 to January 2011, and September 2011 to January 2012. RDS was utilized to recruit the study participants. The details of the recruitment methods have been reported previously [16]. Participants were eligible if they were male, 18 years age or older, a Chongqing resident, had a history of having sex with another man in the past 12 months (sex can be defined as oral, anal, or mutual masturbation), had a valid study recruitment coupon, never participated in the survey previously, and was able to provide written informed consent. The study was approved by the Committees for Human Research of the National Center for AIDS of the China Center for Disease Control and Prevention, Vanderbilt University and the University of California San Francisco. A computer-assisted, interviewer-administrated questionnaire (CASI) was used to collect information including demographic information and sexual behaviors.

### 2.2. Measures

After eligibility screening and obtaining informed consent, each participant received a 20–30 minute computer-assisted interview. The questionnaire asked participants about information on demographics, sexual and drug use behaviors, HIV testing history, and stigmatizing and discriminatory attitudes towards people living with HIV/AIDS (PLWHA).

1. Demographics and sexual risk behaviors: demographic questions included age, ethnicity, education, marital status, employment, sexual orientation, and Chongqing residence status. Risk behavior measures included ever engaging in unprotected sexual intercourse, disclosure of sexual orientation, and the number of male and female sexual partners in last 6 months.
2. HIV prevention and HIV testing services: all participants were asked if they had participated in HIV prevention programs including receiving free condom/lubricants, free STI examination and treatment, and HIV testing. Recent testers were defined as participants who received a test for HIV in the preceding year.
3. HIV/AIDS related stigma and discrimination: individual attitudes towards PLWHA were scored by asking participants about their agreement and disagreement (1 = “yes”; 2 = “no”) with 22 statements. The scale was adapted from two pilot investigations conducted in Thailand and Zimbabwe and reported elsewhere [27, 28]. The psychometric measure includes 3 components: shame, blame and social isolation (10 items), discrimination (8 items), and equity (4 items). In our study, we included the first two components in all 3 years (equity section excluded in 2011) and therefore the first two components (18 items) were analyzed in this paper.
4. Laboratory testing: HIV infection was screened with enzyme-linked immunosorbent assay (ELISA; Vironostika HIV Uni-Form plus O, bioMerieux, Holland) and confirmed with Western Blot test (HIV Blot 2.2 WBTM, Genelabs Diagnostics, Singapore). For HIV positives, The BED IgG-capture ELISA (BED-CEIA, Calypte Biomedical Corporation, Rockville, Maryland) was performed for all HIV-seropositive specimens. Syphilis screening was performed by rapid plasma regain (RPR; Shanghai Rongsheng, Shanghai, China) and confirmed with Treponema pallidum particle assay test (TPPA; Fujirebio Inc., Japan).

### 2.3. Statistical Analyses

We excluded the observations of individuals who participated in more than one round and the first observation for repeated individuals was retained in the final dataset. Since we intend to focus on service utilization analysis rather than the trend of use of these services, we believe aggregating data over three years provides a better picture of service coverage among MSM in Chongqing.

Basic demographic, risk characteristics, HIV prevention service utilization, and HIV testing factors were tabulated. For HIV/AIDS stigma and discrimination, scores were calculated (reverse coding items asked in the positive) for each observation within the overall scale. The factors associated with HIV prevention service utilization (three models) and recent testing (one model) were assessed with multivariable logistic regression after univariate analysis. Adjusted odds ratios (AORs) and 95% confidence intervals (95% CIs) were calculated in the multiple logistic regression analysis using a stepwise method. We calculated odds ratios (OR), 95% CIs and* P* values using bivariate logistic regression for each of these analyses. For multivariable analyses logistic regression was used to calculate adjusted odds ratios, 95% CIs, and* P* values. To adjust the enrollment year's impact to service use or the influence changes in time, we used recruitment year as a variable in each model. Statistical Analysis System (SAS 9.3 for Windows; SAS Institute Inc., NC, USA) software was used for all analyses.

## 3. Results

### 3.1. Socio-Demographics of the Study Participants

In total, we removed 163 repeated participants to obtain 1239 unique participants in the final dataset. A total of 510, 485, and 244 participants were retained in each of the three years, respectively (Table 1). The median age of these participants was 23 years old (interquartile (IQR): 21–27 with a range from 18 to 67). Of participants, 69.6% had a college or higher level of educational attainment, nearly two-thirds currently employed with a full time job. In terms of other demographics, 30.0% were college students, more than three-quarters (78.1%) were local residents, and 7.4% reported being married to a woman. In terms of sexual orientation, 70.0% reported their sexual orientation as homosexual, while 23.9% reported being bisexual, and 5.6% being unknown or heterosexual. A quarter (26.3%) of men in this study reported knowing ≥10 MSM and nearly 40% reported having an income of <1000 Yuan (CNY) per month.

### 3.2. Sexual Behaviors

Of participants, 8.5% reported having sex with a female partner in the past six months, 3.6% reported ever seeking sex for money or goods with male partners in the past six months, and 38% reported having unprotected insertive anal sex.

### 3.3. Prevalence of HIV and Syphilis

Regarding STIs, 4.9% were infected with syphilis and 15.4% with HIV (Table 1). HIV prevalence increased from 11.6% in 2009 to 15.3% in 2010 and 23.8% in 2011. BED-CEIA estimated HIV incidence was 12.3 per 100 persons/year (95% CI: 9.2–15.3), increasing from 7.9 in 2009 to 11.9 in 2010 and 18.2 in 2011 per 100 persons/year. Syphilis prevalence was stable at 4% to 5% across all three recruitment years.

### 3.4. Score of Stigmatizing and Discriminatory Attitudes Towards HIV/AIDS

The median score of stigmatizing and discriminatory attitude towards HIV/AIDS (stigma score) of all participants was 7.0 (mean = 5.9, IQR: 3–8) (Table 1). The average stigma score for those who received testing in the past 12 months (5.6) was lower than that for those who did not receive HIV testing (<6.0) (*P* = 0.03). The average stigma score for those who received free condom/lubricants in the past 12 months (5.4) was lower than those who did not receive HIV testing (6.6) (*P* < 0.01). The average stigma score for those who received any prevention services in the past 12 months (5.6) is lower than those who did not receive any prevention services (<6.2) (*P* < 0.01).

### 3.5. Received HIV Prevention and Testing Services

Only forty percent of respondents reported ever receiving condoms and/or lubricants in the past year; less than a third reported always using free condoms and being satisfied with their use, 27.7% reported ever obtained free STI examination or treatment services in the past year, and more than a third never had HIV testing in the past (Table 1). Of the participants who reported where they got free condoms and lubricants, 33.4% were from community based organizations (CBO), followed by family members (20.9%), sex partners (15.4%), and CDC staff (14.5%).

### 3.6. Factors Independently Associated with Receiving Prevention Services

#### 3.6.1. Factors Correlated with Receiving Free Condoms/Lubricants

Participants who enrolled in 2009 (AOR = 0.1, 95% CI: 0.1-0.2, versus 2011), having a full time job (AOR = 1.5, 95% CI: 1.1–2.0), sexual debut with men at younger age (AOR = 0.7, 95% CI: 0.5–0.9, 9–29 yrs versus ≤18 yrs; AOR = 0.3, 95% CI: 0.2–0.6; ≥30  yrs versus ≤18  yrs), and lower stigma score (AOR = 0.9, 95% CI: 0.9-1.0,OR value per scale point) were more likely to have ever obtained free condoms/lubricants in the last year Table 2, Model 1.

#### 3.6.2. Factors Correlated with Receiving Free STI Services

Participants who enrolled in 2009 (AOR = 0.5, 95% CI: 0.3–0.8, versus 2011), who had larger social networks (AOR = 1.6, 95% CI: 1.2–2.2), those more likely to receive free STI services, those who had unprotected sex (AOR = 0.7, 95% CI: 0.5–0.9), and those who were HIV positive (AOR = 0.6, 95% CI: 0.4–0.9) were less likely to receive free STI services in the past year Table 2, Model 3.

#### 3.6.3. Factors Correlated with Receiving Any of HIV Prevention Services

Participants who enrolled in 2010 (AOR = 1.6, 95% CI: 1.2–2.1, versus 2009), who had higher income (AOR = 1.5, 95% CI: 1.1–1.9; >1000 Yuan monthly), those with larger social networks (AOR = 1.6, 95% CI: 1.2–2.1), and those with sexual debut with men at a younger age (AOR = 0.7, 95% CI: 0.5–0.9; 9–29 yrs versus ≤ 18 yrs; AOR = 0.4, 95% CI: 0.2–0.6; ≥ 30 yrs versus ≤ 18 yrs) were more likely to have ever received any of the HIV prevention services in the past year Table 2, Model 4.

### 3.7. Factors Independently Associated with HIV Testing

Having larger social networks (AOR = 1.8, 95% CI: 1.3–2.4) and lower stigma scores (AOR = 0.9, 95% CI: 0.9-1.0), enrolling in 2010, having higher income (AOR = 1.7, 95% CI: 1.2–2.2; >1000 Yuan monthly), and being HIV-seronegative (AOR = 0.5, 95% CI: 0.3–0.8) were independently associated with receiving a test for HIV in the past year.

### 3.8. Barriers and Facilitators of HIV Testing

**Figure 1:**
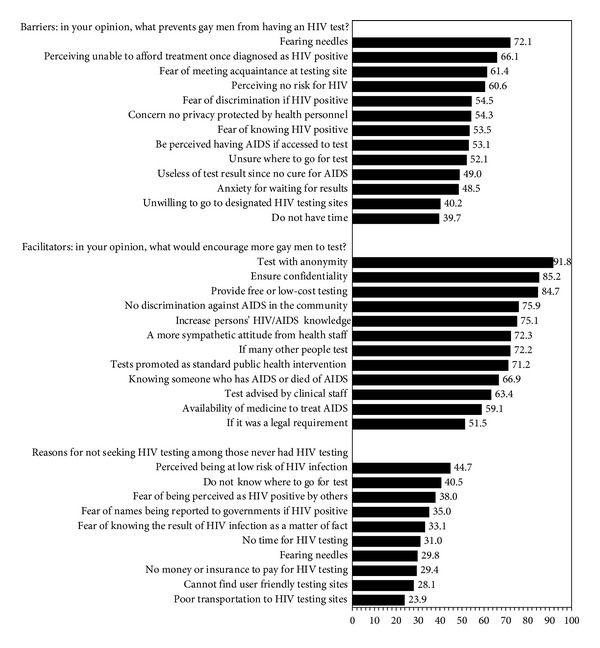
Barriers and facilitators for HIV testing and reasons for not seeking HIV testing.

Of participants, 72.1% reported “fearing needles” as the reason for not seeking a test for HIV, 66.1% perceived they were unable to afford treatment once diagnosed as HIV positive, and 61.4% feared meeting acquaintances at testing sites (61.4%) as reasons for not seeking an HIV test(Figure 1). While nearly two-thirds (60.6%) of respondents perceived no risk for HIV infection, 54.5% feared knowing their HIV serostatus, 54.3% feared to be tested due to discrimination or privacy concerns and 52.1% did not know where to get an HIV test. Regarding facilitators for HIV testing, the majority of respondents (91.8%) choose “test with anonymity,” assurance of confidentiality (85.2%), “free or low-cost testing” (84.7%), less discrimination (75.9%), HIV/AIDS knowledge awareness, and more sympathetic attitude from health professionals (75.1%), as well as promoting HIV testing as part of standard HIV intervention (71.2%) as reasons to have an HIV test. More than half (66.9%) of respondents believed knowing somebody who had AIDS or died of AIDS might be a facilitator for HIV testing uptake.

## 4. Discussion

Statistics from the Chinese Ministry of Health and UNAIDS have shown a worrisome trend in the HIV epidemic among MSM in China [3, 10]. Nationally, the proportion of cases attributed to MSM increased approximately 5-fold from 2.5% in 2006 to 13.7% in 2011 [3]. This suggests that the HIV epidemic is expanding rapidly in this population and that more effective prevention measures are urgently needed. The present study presents a potentially worrying scenario of rapid HIV expansion among MSM in Chongqing, with an alarming HIV prevalence, about 3 times of the national average [12] and a worrisome level of high HIV incidence. Just over half of respondents in Chongqing received at least one HIV prevention service recently, including free condoms/lubricants, free HIV testing, and free STIs examination in the last year. Only a quarter of MSM had a test for HIV recently, while a third had never tested for HIV. This study highlights that HIV/AIDS related stigmatizing and discriminatory attitudes and a variety of barriers must be addressed in order to improve the delivery of prevention services and to expand HIV testing effectively.

Studies [27, 28] have shown that social stigma and punitive civil environments may lead to delays in seeking HIV and STI testing, and subsequent initiation of antiretroviral therapy [18]. Our previous report suggested that stigma and discriminatory attitudes were associated with not seeking HIV testing [28]. The present study suggests that stigma and discrimination are not only associated with HIV testing but are also associated with HIV prevention services, especially the use of free condom/lubricants. This is consistent with the finding from the barriers and facilitators assessment in present study. More than half of participants listed “fear of meeting acquaintance at testing site,” “fear of discrimination if being HIV positive,” and “perceived to be living with HIV/AIDS if initially take HIV test,” as discrimination and stigma related barriers. A recent qualitative study in China suggested that reducing HIV-related stigma and discrimination can actually increase HIV testing and relevant HIV-service uptake [29].

The present study also found the two main facilitators for HIV testing are “testing with anonymity” followed by “assurance of confidentiality.” This finding is consistent with another study that those recruited via social networks (who benefit developing the supportive relationships from group membership) prefer to maintain their anonymity [30]. Unwillingness to go to an HIV clinic was an additional barrier to testing. Financial assistance, transportation support, and mobile testing vans should be considered to address this situation, for example, conducting HIV testing in community venues frequented by MSM. For persons using clinic-based testing, high quality counseling, and nonjudgmental friendly care and service providers can help clients reduce their fear and concerns and facilitate the linkage of seropositive persons to care. Along with expanded education and social marketing, a welcoming and nonjudgmental environment for HIV testing is needed [31].

The barriers and facilitators assessment revealed that fear of needles was the first perceived barrier against taking an HIV test, followed by fear of cost of testing. This finding supports the use of the rapid oral test (ROT) or other noninvasive HIV testing method for testing promotion. China National Guideline for Detection of HIV/AIDS in 2009 [32] recommended saliva based oral rapid HIV testing for use in clinics or Emergency Department. It is necessary to provide training for the use of ROT in community based organizations (CBO) or in grass-root level health agencies.

More than half of the participants reported one of the barriers to HIV testing as not knowing where to get a test. Another important barrier was the perception of being not able to afford HIV testing and/or treatment. This finding further underscored the gap in promoting HIV testing among MSM [33, 34] and the needs to address various barriers in delivering prevention and HIV testing services.

The age of participants in the present study is younger compared with other reports [29, 35], while one predictor for free condom/lubricants accessibility in last year was sexual debut with men at a younger age. Similarly with Choi's study [5], the present study found that the younger age of sexual debut with men was associated with larger size of social networks (*r* = −0.15) and larger social networks were associated with the use of HIV prevention services. However, our findings differ with another report that suggests MSM sexual debut predicted more frequently change of sex partners and other risk behaviors [36]. The finding of our study leads us to believe that the larger size of an individual's social network might be an advantage for the use of HIV prevention services. While RDS was used to recruit participants for research [37, 38] it could also be applied as a mechanism to deliver intervention services among Chinese MSM as it has been used among intravenous drug users (IDU) [39, 40]. Younger MSM may be more intensely engaging in dynamic social networks where individuals interact often with large numbers of peers. Along with more intensity in dynamic cyber life via emerging media, Internet based social network, for example, “Weibo” (microblog, a “Facebook” like social network in China), QQ, email, and other “text message” or instant messaging “Apps” via mobile phone/Internet could serve as effective channels to promote education message for intervention services delivering.

MSM in our study who had full time jobs were more likely to receive condoms and/or lubricants, which corroborated with a recent report in Beijing that lack of stable employment and stable income leads to vulnerabilities to STI infection [35]. Our study shows MSM were more likely to utilize the services in 2010 compared to 2009 but less likely to use services in 2011 compared to 2009. The reason might be due to the RDS recruitment. Along with the longer referral chains, persons enrolled later, who were more hidden, may have been less likely to be reached by the prevention programs. Another possible reason for the peak of service utilization appearing in 2010 rather than 2011 could be that the China-Global Fund AIDS Program delayed funds disbursement and made CBO face the funds vacuum for almost 1 year period since 4thquarter of 2010 to quarter 3rd of 2011 [41]. The delay exposed vulnerability in CBOs' sustainability and lack of monetary incentives resulted in their inactivity in HIV prevention service delivery.

Unprotected receptive anal sex among MSM is the sexual behavior with the highest risk for HIV transmission [42–46]. Nondisclosure of HIV status with casual partners has been associated with sexual transmission. Not knowing a partner's HIV status and not communicating HIV status were particularly common in the casual partnerships. Efforts to improve communication skills to disclose HIV status and use condoms with sexual partners might reduce the sexual transmission of HIV among MSM [47, 48]. However, almost half (48.2%) of the men who tested positive for HIV during the survey were unaware of their infection, highlighting the importance of consistent and correct condom use in the prevention of HIV transmission. Therefore, monitoring the prevalence of risky sexual behaviors and identifying HIV infected individuals who do not know their status could provide valuable information for prevention programs, which are critical to reduce sexual transmission of HIV among MSM.

Multifaceted comprehensive interventions should be implemented. Infrastructure and healthy social norm building (e.g., normalizing condom use and routine HIV testing) should be part of the comprehensive approach. Infrastructure building could be considered such as using mobile vans or equipping health professionals and local CBOs serving local MSM community to promote HIV testing. Healthy social norm building can be achieved by diffusing most relevant health information via available and accessible social media like websites or text messages to promote testing in Chinese MSM [31, 49, 50]. The extent of the risk of acquiring HIV in the United States today is largely defined by a person's sexual network rather than his or her individual behaviors [51]. Group intervention with targeting the behaviors and beliefs from community perspective might be promising as an avenue for prevention in China [52]. For example, collaborating with event promoters offers valuable opportunities to provide condoms, lubricants, and HIV/STI testing [53].

We recognized the limitations of this study. First, self-reported information about the sexual behavior and service uptake may lead to recall bias. Second, because the information was collected by interviewers, some behaviors might have been underreported or over reported. For example, participants might have underreported socially undesirable behaviors (e.g., drug uses) or might have overreported socially desirable behaviors, (e.g., condom use or HIV testing). Third, the nature of the cross-sectional study design precluded the ascertainment of casual relationship. Forth, the study sample comprised of almost one-third college students, which raises the concern of the representativeness of the sample [54]. In addition, the inclusion criteria of willingness to provide a blood specimen may have led to potential under participation in the survey thus increasing selection bias. Despite these limitations, we believe that the present study generated valuable information to enhance the effectiveness of HIV prevention programs among the MSM community.

In summary, the present study revealed that an alarming HIV epidemic is occurring among MSM in Chongqing. Various barriers to the use of HIV prevention service and HIV testing among this population still remain. It is imperative to address the stigmatizing/discriminatory attitudes and other barriers while delivering prevention services and promoting HIV testing.

## Figures and Tables

**Table 1:** Demographics, prevalence of HIV and Syphilis, sexual behaviors, HIV prevention, and testing mehaviors among men who have sex with men in Chongqing, China, 2009–2011.

Factors	N*	%		N*	%
Age (years)			Enrollment year		
18–25	836	67.6	2009	510	41.2
26–35	302	24.4	2010	485	39.1
≥36	98	7.9	2011	244	19.7
Mean (Std.)	25 (6.4)		Unprotected anal insertive sex		
Median	23		No	765	61.7
Range (Q1–Q3)	18–65 (21–27)		Yes	474	38.3
Ethnicity			Free condom use in the last year		
Han	1202	97.4	Never use	49	10.8
Others	32	2.6	Sometime use, unsatisfied	53	11.7
Years of education			Sometime use, satisfied	197	43.6
≤9	100	8.1	Always use, unsatisfied	20	4.4
10–12	277	22.4	Always use, satisfied	133	29.4
>12	862	69.6	Male sex patterns		
Marital status			No anal sex	33	2.7
Single	1099	88.9	Definitely insertive	228	18.5
Married	91	7.4	Mainly insertive	152	12.3
Divorced/widowed	46	3.7	Both	453	36.7
Full-time work			Mainly receptive	230	18.6
No	761	61.4	Definitely receptive	140	11.3
Yes	478	38.6	Stigmatized attitudes towards HIV/AIDS		
Permanent Chongqing residence			Mean (Std.)	5.9 (3.4)	
No	271	21.9	Median	7	
Yes	968	78.1	Range (Q1–Q3)	0–18 (3–8)	
Monthly income in last year			HIV infection		
<1000	479	38.7	No	1048	84.6
1000–2999	542	43.7	Yes	191	15.4
3000–4999	151	12.2	Syphilis infection		
≥5000	67	5.4	No	1178	95.1
Sexual orientation			Yes	61	4.9
Homosexual	872	70.6	Received condom/lubricants in P12M		
Bisexual	295	23.9	No	685	60.5
Others^1^	69	5.6	Yes	447	39.5
No. of MSM you know in Chongqing			Ever had an HIV testing		
≤10	913	73.7	No	422	34.1
≥11	326	26.3	Yes	817	65.9
Had sex with women in P6M			Received free STI services		
No	1134	91.5	No	818	72.3
Yes	105	8.5	Yes	314	27.7
Age at first sex with a man (years)			Getting condom from		
≤18	386	31.2	CBO or peers	251	33.4
19–29	789	63.7	family members	157	20.9
≥30	64	5.2	sex partner/s	116	15.4
			CDC staff	109	14.5
			Medical staff	23	3.1

Note: P6M: the past 6 months; P12M: the past 12 months; STI: sexually transmitted infections; CBO: community-based organization; CDC: Center for Disease Control and Prevention. *Numbers might not add to totals due to missing data. ^1^Others: heterosexual or unknown sexual orientation.

**Table 2:** Factors associated with HIV prevention service and HIV testing.

Independent factors	OR (95% CI)	AOR (95% CI)
Model 1: Factors associated with having ever received condom/lubricants in P12M		
Enrollment year		
2010 versus 2009	0.9 (0.7–1.2)	0.9 (0.7–1.2)
2011 versus 2009	0.1 (0.0-0.1)^†^	0.1 (0.1-0.2)^†^
Fulltime job versus part time job	1.1 (0.9–1.42)	1.5 (1.1–20)^†^
sex debut age with a man (yrs)		
19–29 versus ≤18	0.6 (0.5–0.8)^†^	0.7 (0.5–0.9)^†^
≥30 versus ≤18	0.3 (0.2–0.6)^†^	0.3 (0.2–0.6)^†^
No. of MSM you know in Chongqing (≥11 versus <11)	3.0 (2.3–4.0)^†^	2.3 (1.7–3.1)^†^
Stigmatizing attitudes towards HIV/AIDS*	0.9 (0.9-0.9)^†^	0.9 (0.9-1.0)*

Model 2: Factors associated with having ever receiving HIV testing in P12M		
Enrollment year		
2010 versus 2009	2.6 (2.0–3.4)^†^	2.9 (2.1–4.0)^†^
2011 versus 2009	0.3 (0.2–0.5)^†^	0.5 (0.3–0.8)^†^
Monthly income in P12M		
1000–2999 versus ≤1000 CNY	1.7 (1.3–2.3)^†^	1.7 (1.2–2.3)^†^
3000–4999 versus ≤1000 CNY	1.3 (0.9–2.0)	1.3 (0.9–2.1)
≥5000 versus ≤1000 CNY	0.6 (0.3–1.3)	0.7 (0.3–1.4)
No. of MSM you know in Chongqing (≥11 versus <11)	2.2 (1.7–2.9)^†^	1.8 (1.3–2.4)^†^
Stigmatizing attitudes towards HIV/AIDS*	0.9 (0.9-1.0)*	0.9 (0.9-1.0)*
HIV positive	0.5 (0.3–0.8)^†^	0.5 (0.3–0.8)^†^

Model 3: Factors associated with having ever received free STI services in P12M		
Enrollment year		
2010 versus 2009	1.3 (0.9–1.7)	1.2 (0.9–1.6)
2011 versus 2009	0.6 (0.4–0.8)^†^	0.5 (0.3–0.8)^†^
No. of MSM you know in Chongqing (≥11 versus <11)	1.8 (1.4–2.4)^†^	1.6 (1.2–2.2)^†^
Unprotected insertive anal sex	0.9 (0.7–1.2)	0.7 (0.5–0.9)*
HIV positive	0.6 (0.4–0.8)^†^	0.6 (0.4–0.9)*

Model 4: Factors associated with having ever received any prevention services in P12M		
Enrollment year		
2010 versus 2009	1.6 (1.3–2.1)^†^	1.6 (1.3–2.1)^†^
2011 versus 2009	0.3 (0.2–0.5)^†^	0.4 (0.3–0.5)^†^
Monthly income		
1000–2999 versus ≤1000 CNY	NA	1.5 (1.1–1.9)^†^
3000–4999 versus ≤1000 CNY	NA	1.3 (0.8–1.9)
≥5000 versus ≤1000 CNY	NA	1.6 (0.9–2.9)
No. of MSM you know in Chongqing (≥11 versus <11)	2.1 (1.6–2.7)^†^	1.6 (1.2–2.1)^†^
Sex debut age with a man (yrs)		
19–9 versus ≤18	0.7 (0.6–0.9)^†^	0.7 (0.5–0.9)^†^
≥30 versus ≤18	0.4 (0.2–0.7)^†^	0.4 (0.2–0.6)^†^

Note: OR: Odd ratio; 95% CI: 95% confidence interval; AOR: adjusted odd ratio; P6M: the past 6 months; P12M: the past 12 months; *P < 0.05; ^†^ *P* < 0.01; NA: not available; all the four models were adjusted with sexual orientation, age, married status, education, employment, sex debut age with men, number of male sex partners, stigmatizing/discriminatory attitudes (consecutive variables), HIV infection status, having multiple male sex partner or not, and anal sex pattern (insertive or receptive).
